# Climate change inaction: Cognitive bias influencing managers' decision making on environmental sustainability choices. The role of empathy and morality with the need of an integrated and comprehensive perspective

**DOI:** 10.3389/fpsyg.2023.1130059

**Published:** 2023-03-06

**Authors:** Dario Natale Palmucci, Alberto Ferraris

**Affiliations:** Department of Management, University of Turin, Turin, Italy

**Keywords:** cognitive bias, climate change, empathy, morality, decision making, strategic decision making, managerial psychology, sustainable development

## 1. Introduction

Although it is now universally recognized that climate change will have large and serious consequences not only for the planet but also for business operations, the adaptation of strategic sustainable choices continues to fail. It must be recognized that many people and organizations have already taken some steps in this direction, but “*if a car is heading south, slowing down will not cause the car to head north. Sooner or later you will need to make a 180 degree U-turn*” (Mcdonough and Braungart, 2010). These words effectively explain how a decisive (if not total), trend reversal is necessary to avoid the disastrous consequences that global warming is already causing. In this regard, various disciplines (from engineering to finance, from political sciences to medicine, etc.), are contributing with their knowledge in understanding the causes of this condition of inertia in the implementing of effective solutions. The work that follows represents an attempt to understand why individuals (and in particular managers and organizations), do not implement sustainable behaviors and continue not to consider the climate factor sufficiently in their daily decision-making. The present study is carried out considering the issue of the climate problem from a psychological-managerial point of view, referring to the substantial scientific literature that has focused on the study of cognitive biases (Enke et al., 2021) and their influence in the corporate decision making process regarding environmental sustainability. That said, there are gaps of knowledge in the literature. These gaps refer to the fact that, if on one hand there are several studies focusing on biases and their influence in the decision making (Acciarini et al., 2020) of sustainable actions, from the other hand there are no sufficient works considering biases together with other important elements which could have an impact on this relation. In other words, this stream seems to be fragmented and a comprehensive perspective is missing. For this reason the objective of this conceptual work, as also done in past studies that used a similar approach (Mohammed, 2013), is to shed light on various elements in an integrated perspective. In order to do this, the role of other variables, as the perceived moral intensity of the climate problem and the empathy owned by individuals and managers, will be considered. As will be seen in the following sections, the psychological component is very important for understanding the phenomenon and emphasizing its relevance would guarantee further recognition to the managerial psychology, which would therefore have the opportunity to dialogue with other disciplines to tackle the increasingly complex problem of climate change. One of the great limitations encountered by science in this area has been, indeed, proceeding by “scientific bubbles” and not arriving at a common reference framework that consider all components and embraces all disciplines. Considering all these elements, this paper intends to found a theoretical basis useful for other scholars to keep studying the matter.

## 2. Humans' decision making process

### 2.1. Early studies on decision making

The interest of the scientific community regarding the mechanisms that individuals implement to make their own decisions (Secchi, 2010) is not a recent thing since the first major contributions on the subject were published as early as the middle of the last century. In fact, the beginning of studies on decision making dates back to the mid-fifties, when the main purpose of the research was to describe how an individual should have behaved in order to make decisions in a completely objective and rational way (Edwards, 1954). The first important model in this direction, in fact, is that of ?expected utility” by Von Neumann and Morgenstern (1947). According to the two authors, individuals act in a wholly rational way since the decisions they take are the result of an algebraic elaboration obtained by weighing the utility of each possible result by its probability (a real calculation of the weighted average of the utilities of different results). The individual who finds himself in conditions of uncertainty, therefore, on the basis of a set of axioms which provide him with criteria for making choices in a rational way, manages to calculate the weighted average of the utilities of the various results, and, rationally, will choose the alternative that absolutely offers the highest gains and lowest losses, or, in other words, the one that offers the highest expected utility. This approach, which provides for the optimization of the limited resources available and is based on complete objectivity in the decision-making process and on the rationality of individuals, is questioned in the following years by Simon (1956), which introduces the concept of ?bounded rationality.” In fact, unlike his predecessors, the author sustains that it is not possible for the individual to know all the alternatives of action nor to foresee all the consequences of the decisions taken, since these are sometimes remote and indirect, and that the decision-making process is influenced by important cultural, personal (cognitive, ethical, etc.), as well as temporal (available time) limitations. According to the concept of “limited rationality,” therefore, people have a reduced ability to process all the information (due to the constraints mentioned above), and the outcome of the decision-making process will therefore correspond to taking actions that cannot be defined as optimal, but, less objectively, only “satisfactory.”

### 2.2. The prospect theory and the framing effect of Kahneman and Tversky

A few years later, the fundamental contribution offered by psychologists Kahneman and Tversky with the *prospect theory* (Kahneman and Tversky, 1979), provided a more realistic description of how subjects actually behave when they have to make choices. According to the authors, the decision-maker needs a “perspective” with which to analyze the various outcomes of the choice and two phases can be distinguished to make a decision. A first phase provides for the classification of the acts, results and contingencies, and then there is a concrete evaluation of the various aspects by the individual to make the most optimal decision (Kahneman and Tversky, 1981). In other words, outcomes are simply considered as positive or negative deviations from a neutral cognitive reference point (arbitrarily set by the decision maker) to which a value of zero is assigned, and, if the variation is considered positive the result is evaluated as a gain, if the deviation is considered negative then the result is evaluated as a loss. Nevertheless, as highlighted by Kahneman and Tversky, it is essential that the decision-maker carries out an analysis of the decision-making problem in question, analytically framing the various aspects (what the authors call “*Framing Effect*”—Kahneman and Tversky, 1981). According to this aspect, the context in which a person finds himself making a decision has a decisive impact on the choice itself and depending on how the problem is presented to the individual, the starting point will be different and, consequently, the results of the actions will be different. This contribution, in addition to demonstrate that human beings violate the principles of economic rationality, also suggests that, although subjective values differ among individuals, losses take on a greater value for the subject than gains. With reference to this finding, Tversky and Kahneman propose the value function as an “S”-shaped graph (which explains that if the reference point is determined in such a way that a given result is considered as a gain, then the decision maker will tend to make non-risky choices; on the contrary, in the event that this reference point highlights an outcome in terms of loss, then the decision maker will tend to make risky decisions—Kahneman and Tversky, 1979).

To conclude, Kahneman and Tversky's findings showed that individuals make decisions not in a rational way, but by using some cognitive mental procedures that allow them to make decisions according to the complexity of the situation and the limitations of its information storage and processing system. In other words for Tversky and Kahneman, to assess probabilities, to predict values and to make judgments under conditions of uncertainty, people are not using sophisticated rational processes, but a limited number of mental shortcuts: the heuristics. These findings have been fundamental for the birth of the concept of cognitive bias.

## 3. Managerial applications of cognitive biases

### 3.1. Cognitive biases and organizational/strategic decision-making

As seen in the previous paragraph, the contribution of Tversky and Kahneman was as revolutionary as it was fundamental to arrive at the concept of Cognitive Bias (Enke et al., 2021). Regarding the existence of an unambiguous definition of cognitive bias, there does not seem to be one universally and transversally recognized but all converge describing these as systematic errors of judgment and deviations from the norm and from rationality (Haselton and Buss, 2000). More precisely with reference to the topic of this work, cognitive biases (Enke et al., 2021) are ever present element in the decision-making process of people, and, similarly, they are also present in the strategic decision making process of managers (Das and Teng, 1999). In fact, biases are very frequent in the strategic decision making process (Mcfadden, 2022) with managers who are often required to make decisions in complex situations with complexity and scarce knowledge of all relevant components and facts to make a decisions in the various aspects related to the corporate world (Keh et al., 2002). For example, these biases can lead to systematic errors and low chances of survival for new ventures (Cooper et al., 1988; Hayward et al., 2006). For these reasons, most of the studies have focused on the negative influence of biases on the strategic decision making process (Mcfadden, 2022) and how these can jeopardize it. In this direction, (Das and Teng, 1999) have analyzed the most salient relationships between the 5 main modalities of strategic decision-making and 4 certain cognitive biases (a priori hypothesis and focus on limited objectives; exposure to limited alternatives; sensitivity to probabilities of outcome; illusion of manageability) highlighting that biases can negatively affect all modalities and processes of the strategic decision making (Acciarini et al., 2020). That said, the contributions of the scientific community on the influence of cognitive biases on the decision-making process (Leicht-Deobald et al., 2022) have multiplied in recent years, and, to date, this area of study has become very relevant (Gregoire et al., 2011) and the contribution of Das and Tend has been joined by many others. However, the main focus of this work is on those biases impacting the managerial decision making of sustainable conducts. For these reasons in the next paragraph a list of cognitive biases (Enke et al., 2021) influencing people and managers' decision making on environmental sustainability choices will be deeply analyzed and addressed.

### 3.2. Bias related to environmental sustainability choices

From what has been analyzed, it is clear that cognitive biases of managers affect the survival of the organizations they are part of (Gudmundsson and Lechner, 2013) and it can therefore be said that managers vary in their cognitive composition and this leads to a significant influence on the company and its success. Furthermore, just as the decision-making process presents numerous cognitive biases (or prejudices), due to the high degree of uncertainty that this process foresees, it is important to underline how these prejudices are also relevant in the decisions concerning the environmental actions that are undertaken by people (Hoffman and Bazerman, 2007), which is the main focus of the present study. This does not mean that the psychological component is the only one to take into consideration in understanding why people and managers do not act in a truly sustainable way. In fact, it is important to remember that unsustainable behavior mainly occurs due to the presence of structural barriers, such as the presence of infrastructures adverse to the climate, or the low average income which limits the possibility of people and businesses to buy too expensive solar panels for example, but also living in a rural area which is often poorly served by public transport and so there is no alternative to using a personal car and so on (Gifford, 2011). That said, there is also another aspect that prevents subjects from actually carrying out actions that would facilitate mitigation, adaptation and environmental sustainability (i.e., the psychological aspect) which goes beyond the control of the individual himself. Table 1 below, shows a list of the main biases (and reference literature) which, according to the studies analyzed, have an influence on decision-making and the implementation of pro-environmental behaviors.

**Table 1:** List of the main biases which have an influence on the decision-making regarding the implementation of pro-environmental behaviors.

**Bias**	**Description**	**Key literature**
Present bias and discount the future	Tendency not to consider the long-term returns of investments aimed at preventing climate change which, therefore, are discarded as the short-term return is perceived as inadequate and as the results in terms of savings and/or earnings are only visible after decades. In other words, since companies need to demonstrate the return on investment on a quarterly or at most annual basis, pro-environmental investments (whose economic advantage is already uncertain in itself), which guarantee returns only after 1 or 2 decades, cannot be preferred to other traditional investments whose short-term return is higher	Shu and Bazerman (2010), Mazutis and Eckardt (2017) Gifford (2011), Weber (2017), Newell and Pitman (2010)
Bias of diffusion of responsibility, comparison with others and egocentrism	Tendency to think (by developed countries), that the problem of climate change will not be solved until developing countries put the brakes on their population growth and really start collaborating internationally, intervening and respecting the agreements made on the climate. In other words there is the tendency to think that the main responsibility for the problem lies mainly with them. On the side of developing countries, however, there is a tendency to think that climate change is a consequence of the industrialization of the past implemented by Western countries and of how they have exploited resources, in addition to their consumer habits of the present. This bias is also present at a *sector level*, with tendency to think that if other sectors or companies do not work as they should to solve the environmental problem, everyone might as well think about procuring as many resources to do their business	Shu and Bazerman (2010), Mazutis and Eckardt (2017), Gifford (2011), Engler et al. (2019), Newell and Pitman (2010)
Planning fallacy bias	Tendency to underestimate the times and costs of projects (in the environmental field, but also other types of interventions) which in the end turn out to be more wasteful and expensive than initially estimated. This trend, combined with the uncertainty of pro-environmental investments which, being new, involve a greater risk margin, would seem to discourage the decision-making and implementation of environmental sustainability projects.	Singh and Ryvola (2018), Gifford (2011)
Confirmation bias	Tendency to only consider information that is in line with one's thinking, rejecting any information that would lead to having to change one's mind or to change one's behavior. This causes people to remain skeptical of information campaigns and convinced that man alone cannot do much to counter global warming	Newell and Pitman (2010)
Status-Quo bias, risk aversion and resistance to change	Tendency to think that before embarking on new paths and investing in environmental sustainability projects, it is better to make what already exists and fully exploit what has already been invested in. Tendency to think that with regard to environmental sustainability projects, where little is known about them yet, the risks seem to be greater than the benefits. Furthermore, since to deal with “something new” will necessarily mean to proceed by attempts with a high risk of failure of the very first projects and the need to quickly redirect to new alternative projects to try, there is the tendency to think that it is better avoid such risks. In summary, it is the tendency to think that if you had to invest in something that didn't go well, it would all have been a waste of time	Godefroid et al. (2022), Singh and Ryvola (2018), Mazutis and Eckardt (2017), Gifford (2011), Weber (2017), Engler et al. (2019), Arvai et al. (2012), Newell and Pitman (2010)
Availability bias and thought shortcuts	Tendency to consider events available in memory more probable. In other words, if people have never directly experienced a high-intensity catastrophic climatic event (for example a flood, a tornado, a severe drought, etc.), they are more inclined to think that these things will never happen and probably this is the reason that does not push decision makers to implement sustainable behaviors and really invest to protect the environment	Singh and Ryvola (2018), Mazutis and Eckardt (2017), Arvai (2012), Newell and Pitman (2010)
Single action bias and illusion of being right	Tendency to overestimate the contribution of a pro-environmental action that is implemented (ignoring a series of other actions that have an impact on the environment but that has been decided not to change), mistakenly thinking that the impact on the environment with the implementation of this one unique (or few) actions. It is the tendency to think that putting into practice only some of the pro-environmental actions (for example recycling and properly disposing of waste, respecting gas emission limits, etc.), can compensate for all those other actions which unfortunately cannot be discarded and which have yet to be done in the traditional way	Singh and Ryvola (2018), Gifford (2011), Holmgren et al. (2022)
Framing effect bias	Not perceiving the term “climate change” as it is presented as something really serious or catastrophic and therefore as a really urgent problem (as it would be if it were presented as an “environmental catastrophe” or as “environmental abuse”). Even the images that are often seen on the consequences caused by global warming are not perceived as serious and do not arouse a sense of urgency and urgent danger	Mazutis and Eckardt (2017), Newell and Pitman (2010)
Anthropocentrism bias	Difficulty in conceiving that in order to try to solve the problem of climate change, the human being should first of all be able to take a step back and see himself on a par with all other species as a whole in contact with nature. The tendency, on the other hand, seems to be to think solely that in order to try to solve the problem of climate change, human beings should first of all focus on their ability to implement sustainable development—Ensuring that their present needs are met without compromising the future generations the possibility of satisfying their own, demonstrating that they put only humans (and his descendants), at the center of everything	Naudè (2017)
Will bias	Tendency to think that simply respecting the rules that the institutions (United Nations, European Union, etc.), impose on the maximum emissions allowed, or on waste disposal, etc., means already doing enough or at least everything that is feasible to prevent environmental disasters caused by climate change. In other words, the task of managers is and remains mainly that of maximizing the interests of the company in compliance with the rules imposed by the regulatory bodies	Mazutis and Eckardt (2017)
Anchor effect bias	Tendency to underestimate the consequences that a 2–5 degree Celsius increase in global temperature can have on oneself and on the environment. The 2–5 degrees become an anchor of reference and it is thought that the problem is not serious given that in the alternation of the seasons (between summer and winter), the fluctuation of degrees is greater and it is sufficient to use air conditioning to solve the problem	Mazutis and Eckardt (2017), Newell and Pitman (2010)
Optimism bias	Tendency to think that a technological solution to the problem of climate change will surely be found. Somehow science and technology will manage to contain the consequences of global warming as it happened for other problems faced by man in the past. Tendency to think that the catastrophic consequences of the warming of the earth's crust will not be seen firsthand because they will only happen in 20–25 years (or that they are more likely to happen in other places in the world)	Shu and Bazerman (2010), Mazutis and Eckardt (2017), Gifford (2011), Chadee et al. (2021)

Source: Personal elaboration.

## 4. The key role of the moral intensity of the climate problem

As highlighted in the previous paragraph, cognitive biases have a negative influence on pro-environmental behaviors and decisions of people, managers and decision makers in general (Enke et al., 2021) in the organizational/institutional sphere. It is also important to underline that, from the literature analysis, it emerged that the perceived moral intensity of the climate problem of people and managers play a fundamental role in explaining under what conditions behaviors and decisions to contrast global warming are put in place. As stated by Mazutis and Eckardt (2017) in fact, cognitive biases negatively influence the decision making of sustainable behaviors and moral intensity acts as a relevant mechanism for the ethical and environmental choices to be made. A crucial aspect of their study is based on the belief that the problem of climate change could be seriously tackled and contrasted if one began to attribute to it the connotation of a real moral issue which, in addition to harming other subjects, also violates people's rights. Therefore, according to them, problems that have a high level of moral intensity lead subjects to implement more sophisticated moral reasoning processes than issues with low moral intensity. The main cause of this “inaction” by companies therefore lies in the fact that people and key decision makers fail to perceive “climate change” as a moral issue due to the cognitive biases that govern traditional decision-making models in organizations. This occurs because, if the moral intensity of a problem is low (as it is in the case of climate change), cognitive biases prevent decision makers from recognizing the problem as pressing and important and, therefore, as requiring immediate strategic choices. On the contrary, if it is perceived that a problem is characterized by a high level of moral intensity, the same moral intensity is able to penetrate the barriers established by the biases managing to give concrete answers at any moment of the decision making process. Also Detert and colleagues (2008), in their study called “*Moral Disengagement in Ethical Decision Making: a study of antecedents and outcomes*,” they seem to go in the same direction by hypothesizing and finding that not perceiving about a morally important issue increases the likelihood that people will make unethical decisions. Another very important contribution in this direction is given by Dukerich et al. (2000) with their “*Moral Intensity and Managerial Problem solving*.” These authors, on the basis of Jones theoretical model of ethical decision making (Jones, 1991), focused on the role of the moral intensity of the problem in the decision making process of managers and, above all, on the promptness with which managers respond to problems that are perceived as highly moral compared to those who are not equally so. According to these studies therefore, different managers seem to perceive and morally categorize the same problem differently especially if the surrounding of the problem and the actions to be taken are uncertain (such as those concerning the problem of climate change). In other words, the readiness with which decision makers take action is influenced by the degree to which they perceive a problem (and the consequences that could derive from it), and the decision as moral.

### 4.1. The role of empathy

From what was highlighted in the previous paragraph, the perceived moral intensity of the climate problem plays a fundamental role in explaining the conditions in which the behaviors and decisions to contrast global warming are implemented by managers. The same seems to apply to the degree of empathy possessed by the decision makers and it is therefore important to analyze in detail the works done for better understanding the relationship between this and the behavioral propensity of people and managers. More Specifically, there are many contributions in the literature that have addressed the study of empathy and, therefore, there are many definitions of empathy that have followed one another in recent years. Among these, an example is the definition given by Berenguer (2010), who in his work describes empathy as that emotional response that allows people to perceive the psychological state of the others (to put themselves in their shoes). Also interesting is the definition of Batson (2009) which defines empathy as the ability of an individual to understand the perceptions of others toward something. Beyond the various definitions, the focus in this work is to try to understand how this can be related to the pro-environment behaviors/decisions of decision makers in organizations, arriving at the creation of a sort of “environmental empathy” (as define it Islam et al., 2018) in their study of the hospitality sector. Specifically, these researchers, studying the relationship between workers' pro-environmental behaviors, identification with their company and the corporate social responsibility policies of the organization to which they belong, found an interesting moderating role of empathy in the relationship between these. In particular, the authors, in agreement with what Detert and colleagues stated in a previous contribution (Detert et al., 2008), show that empathy morally involves workers and that this would help them in decision-making at an ethical level and in ethical behavior.

Islam and colleagues also find that a greater degree of empathy involves greater attention to pro-environmental behaviors and decisions and that empathy and ethical behavior would also be reflected outside the work context. On the other hand, individuals with low levels of empathy have a lower ability to perceive the conditions of their environment (even when the environment affects the people themselves with intense catastrophic events) and consequently implement less sustainable behaviors. This study is not the only one showing that workers with greater empathy behave in a more sustainable way because they perceive the conditions of their territory more, as if they developed a sort of environmental empathy. In fact, Tian and Robertson (2017), in their study carried out on about 200 bosses and workers, found that those individuals with greater empathy are the ones who voluntarily implement more than other pro-environmental behaviors. Also Berenguer (2010), with his study called “the effect of empathy in environmental moral reasoning,” found a relationship between empathy, values, emotions and decision making at the level of environmental sustainability and, even in Batson's model of altruism (Batson et al., 2002), it is stated that higher levels of empathy correspond to greater pro-environmental attitudes (and therefore behaviors).

### 4.2. The need of an integrated and comprehensive perspective

This work highlights an important gap in the literature: the effect of cognitive biases on economic decision making (Acciarini et al., 2020) has been largely studied in the field of behavioral economics but never considering different components in an integrative perspective (bias, moral intensity of the issue, grade of empathy of the decision makers, etc.). In other words, there is a dearth of research focusing on how all these different components influence together the way individuals and managers perceive the environmental problem and act accordingly (Gifford, 2011). This inability to see the problem in an integrated scheme contributes to the scarce consideration given to the managerial psychology as a relevant and useful discipline to contribute understanding how to increase behaviors for the protection of the environment. The literature review performed in this piece of research confirms that biases are often present in the decision-making process (Acciarini et al., 2020) of environmental sustainability choices and, along with other individual components (scarce perceived moral intensity of the climate problem and low empathy owned by managers), influence the propensity of people and managers to act sustainably. In particular the following propositions are proposed:

### 4.3. Relationship: Cognitive bias—pro-environmental behaviors/decisions

Cognitive biases influence the decision making process and the implementation of sustainable behaviors by managers. In particular:

a1) A high propensity of managers to cognitive biases decreases the probability that they implement pro-environmental behaviors and make decisions taking into consideration the climate problem;

a2) A low propensity to cognitive biases (or absence) allows managers to implement pro-environmental behaviors more easily and above all to take the climate aspect into strong consideration in their strategic decision making process.

### 4.4. Role of the perceived moral intensity of the problem

Not all managers consider the climate problem with the same scale of seriousness and relevance. This also depends on the degree of perceived moral intensity regarding the environmental issue, which influences the relationship between cognitive biases and sustainable decisions/behaviors as follows:

b1) When managers perceive the climate problem as morally relevant, they implement a greater number of pro-environmental behaviors and the negative effect of cognitive biases on decisions regarding environmental sustainability decreases. The high moral intensity attributed to the climate problem generates a sense of urgency and this affects the timeliness with which managers act in favor of the environment;

b2) When managers do not attribute seriousness to the climate problem from a moral point of view, the negative effect of cognitive biases on decisions regarding environmental sustainability is stronger. The low moral intensity attributed to the climate problem means that the sense of urgency is not generated and, consequently, managers tend not to give priority to pro-environmental behavior and investments, limiting themselves to simple compliance with the rules and limitations imposed by the regulatory authorities.

### 4.5. Role of empathy/environmental empathy

Managers show different levels of empathy (or environmental empathy) and this plays an important role in the relationship between cognitive biases and sustainable decisions/behaviors. In particular:

c1) A high degree of empathy possessed by managers mitigate the negative effect of cognitive biases on the implementation of sustainable choices. The managers who find it easier to put themselves in other people's shoes and be able to perceive the environment around them, in fact, go far beyond the rules imposed by the regulatory bodies and in the decision-making process demonstrate that they are creatively and proactively committed in implementing the practices of environmental sustainability in all phases of production of one's company or in the operations of one's business, despite the difficulties and the difficult context in which they find themselves operating;

c2) In the case of managers with a low degree of empathy (both toward other people and toward the surrounding environment) the negative effect of cognitive biases on the implementation of sustainable choices is not mitigated and, consequently, the managers do not prove to be proactive toward the climate problem and are limited to a simple respect for the limitations imposed by the regulatory bodies.

## 5. Implications, limitations, and future research

### 5.1. Implications

There is wide a individuals' tendency to make errors during their decision-making process and research shows the presence of this tendency in many organizational contexts (Stelmakh et al., 2019). As seen, biases prevent managers from making rational choices in many areas within companies and environmental sustainability choices are not excluded. As analyzed in the previous sections, also moral intensity given to the climate problem and environmental empathy play a key role and what this conceptual paper has reviewed and proposed is relevant at different levels. In particular, this work provides four implications. In the first instance, it contributes to the literature on decision making by classifying the biases that affect individuals in their environmental sustainable decisions. This means that, the present study, based on a solid basis of scientific knowledge obtained thanks to the literature review, explains how individuals, decision makers in organizations and institutions make environmental sustainability decisions and implement pro-environmental behavior. In particular on a theoretical level, this conceptual work considers together more elements (*cognitive biases—moral intensity of the environmental problem—empathy)* contributing to the dearth of research in analyzing the topic with a comprehensive perspective.

Second, at a managerial level, organizations may use this article to promote awareness on the mechanisms that can affect sustainable decisions and to invest in enhancing their culture of sustainability. This means they should try to promote organizational cultures that give greater prominence to the climate problem and, above all, raise awareness of the seriousness of the situation and the moral component of the environmental issue. The study shows, in fact, that managers who do not recognize the problem as having a moral impact limit themselves to simple compliance with the rules and do not face it proactively. In addition, at a managerial level, the implications of the study are significant for the human resources function (Leicht-Deobald et al., 2022) within organizations and companies should focus in aligning the HR Management function to the new needs (Picone et al., 2021). In particular, the HR area that can benefit from what has been analyzed is the *learning & development*, since the HR training departments in the companies are affected for several aspects. First of all, regarding the achievement of an adequate level of *sustainable leadership* (which represents a fundamental shift of mentality for companies and for societies - Smith and Sharicz, 2011), the results of the present study could be incorporated into any *leadership development* program. Furthermore, even at a general level, companies can and should definitely invest resources in training programs and time to limit the negative impact of biases on individuals' sustainable behavior. In fact, as showed by Lilienfeld et al. (2009), few e-learning sessions (as educational video and computer games) are enough to improve people's ability to reduce their biases and such approach can be easily adopted in the organizational contexts. Third, the paper provide suggestions also to the recruiting and career management departments as, if it is true that the most empathetic subjects are those who are more likely to consider the climate factor as a priority in decision-making, then it becomes a duty for companies to ensure that this important attitude of the human being is present in the organization and that selection, promotion and career advancement initiatives take this aspect into account (and this would guarantee the company the constant presence of managers who are increasingly sensitive to the issue of environmental sustainability). Fourth, the findings of the present study may be relevant for policy makers and should be considered by governments and supranational regulators seeking to implement policies on climate change and which aim to encourage environmentally responsible behavior, as they provide the tools to make more complete and informed decision making. The biases and their consequences described in the study represent opportunities to improve the way information about climate change is presented to individuals. Furthermore, it is important to raise people's awareness of the issue of climate change and arouse a sense of urgency toward the problem, because only by acting promptly it is possible to mitigate the consequences and curb the dramatic advance of global warming (Shome and Marx, 2009). Policy makers therefore cannot fail to take into consideration the psychological aspect in solving the problem since, as emerges from the present study, it is essential to know how people make decisions, to be aware that cognitive biases influence the engaged in pro-environmental behavior and knowing that the most efficient way to mitigate the effects of cognitive biases related to climate change is to recognize it as a moral problem, intensifying the magnitude of the consequences, the social consensus, the probability of the effect, the temporal immediacy of the consequences, the proximity with the problem and the concentration of the effect of global warming (Mazutis and Eckardt, 2017).

### 5.2. Limitations and future research

More specifically, this article discussed the cognitive biases that can prevent the implementation of pro-environmental behaviors, along with the role played by perceived moral intensity of the environmental problem and grade of empathy owned by individuals in general, and managers in particular. Given the importance of the global warming problem, the documented relevance of cognitive biases affecting the judgments and behaviors of individuals and managers and the role played by empathy/perceived moral intensity of the issue, the value of this work must be recognized for having considered all these components together. That said, the present study has limitations and future research is needed to arrive at a better understanding of the phenomenon under study. In fact, on the other hand, it is important to remark that, in particular methodologically, the existence of a unique integrated framework should be confirmed quantitatively. As said, the study has considered various variables together, but a single reference model quantitatively validated that includes all the variables involved in the relationship between *cognitive biases and pro-environmental behavior* (and that is recognized by most of the scientific community committed to studying the barriers that prevent sustainable decisions from being made) is still missing. The elaboration of a shared model could constitute a solid basis on which to start a discussion table also with experts from other disciplines who face the problem of climate change on a daily basis (Gifford, 2011). In other words, in order to provide the reference literature with a valid and useful contribution to the explanation of the phenomenon, the present mainly conceptual and exploratory study, should be suitably integrated at a quantitative level. On this direction, regarding the pro-environment behaviors implemented by managers of companies, for example, future studies could use quantitative indicators such as the *SETAC Life Cycle Assessment* (LCA) of the Society of Environmental Toxicology and Chemistry^1^, which is a method of assessing the environmental impact associated with the production of a good or an activity, through the identification of the quality and quantity of energy, materials used and waste released into the environment. The estimate examines the entire life cycle of a product, process or activity (starting from the extraction and processing of raw materials to recycling and disposal). A further quantitative indicator that can be used to study the sustainability of buildings is the *Leadership in Energy and Environmental Design* (LEED) of the USGBC^2^(U.S. Green Building Council). A company that has LEED is attentive to the environmental impact and includes the sustainability factor in its decision making. Also the propensity to bias and the component of moral intensity related to the climate problem should and could be investigated quantitatively and in an integrated way (building on previous studies that have used objective and quantitative measures of these components but in isolation, for example). Still with regard to the measurement of moral intensity, another limitation of the present study (and also of previous studies that investigated moral intensity in managerial decision-making), is that of not differentiating between the various components of moral intensity (Jones, 1991), which according to Jones is a multidimensional construct (example: probability of the effect, social consensus, etc.). A further level of analysis could be to distinguish between the moral intensity of the problem at the collective level (with one's own group) and the moral intensity of the problem at the individual level. A further suggested starting point for the investigation could also be to analyze the differences between managers who work in non-profit organizations engaged in humanitarian work and those who work in profit companies. Furthermore, additional control variables to be included in a following quantitative study could focus on gender, age, educational level, cultural context and level of decision-making power which are all factors that certainly deserves more attention. Finally, it would be interesting to investigate the existence of a direct relation between empathy (or environmental empathy toward the environment as Islam and colleagues define it—Islam et al., 2018) and moral intensity of the issue, in order to understand in which conditions a greater degree of empathy leads to greater attention toward pro-environment behaviors and decisions (and if empathy and ethical behavior would also be reflected outside the working context—Detert et al., 2008).

## 6. Conclusion

In an increasingly uncertain era and globalized market, environmental sustainability is one of the key elements for organizations efficiency and countries' development, and essential to ensure their continuing competitiveness and survival (Bresciani et al., 2021). The objective of the present study was to understand why companies and managers, despite being aware of the enormous negative consequences that climate change will cause, often continue not to adjust their strategic decision-making processes toward a more “green” and sustainable approach. With this premise, an attempt was made to look at the phenomenon from a managerial psychological point of view and therefore the three variables (*cognitive biases—moral intensity of the environmental problem—empathy)* were put together, and a consistent review of the recent literature was analyzed in order to understand the influence of these variables on the environmental sustainability choices implemented by people, managers, companies and institutions. Thanks to the analysis of the literature carried out, it is possible to conclude that:

*A)* Managers who, in making decisions, have little consideration of the environmental factor and therefore tend to implement pro-environmental behavior to a lesser extent, seem to have a strong propensity to cognitive bias, a reduced perception of the moral intensity of the climate problem and a lower grade of empathy; *B)* Decision makers whose personal-managerial profiles are well endowed with an intense moral perception of the climate problem, little propensity to bias and higher level of empathy, are the ones who mostly make pro-environmental decisions and implement sustainable behaviors in the daily performance of their role. This is translated into a proactive, creative and continuous implementation of pro-environment behaviors. In other words, these managers demonstrate that they go far beyond simple compliance with the laws imposed by regulatory bodies, and they are committed on a daily basis to reducing the environmental impact of their company's operations at each production stage. This contribution is useful for anyone who wants to further expand the knowledge on the topic and analyze the reasons that drive managers not to behave in a sustainable way, finally taking into consideration the managerial psychological aspect of the climate problem as well.

## Author contributions

DP: abstract, main body, central part, and conclusions. AF: abstract, introduction, conclusion, and tables. Both authors contributed to the article and approved the submitted version.

## Footnotes

^1^ https://www.setac.org/

^2^ https://www.usgbc.org/
